# The Test for Milk

**Published:** 1903-09

**Authors:** 


### The Test for Milk.

In the discussion of the best means of securing pure milk for Chicago, and the best way for families to test the supply, one wise fellow says: "Let the milk stand in a warm room. If it does not turn sour it contains preservatives and should not be used." That's all right. If it doesn't turn sour it is unfit for use, and, of course, it's unfit for use if it does turn sour.—*Chicago Clinic.*

